# 3D Printed Cellulose-Based Filaments—Processing and Mechanical Properties

**DOI:** 10.3390/ma15196582

**Published:** 2022-09-22

**Authors:** Julia Utz, Jokin Zubizarreta, Nico Geis, Kirsi Immonen, Heli Kangas, Holger Ruckdäschel

**Affiliations:** 1 Department of Polymer Engineering, University of Bayreuth, Universitätsstraße 30, 95447 Bayreuth, Germany; 2 VTT Technical Research Centre of Finland Ltd., Tietotie 4E, FI-02044 VTT, FI-02150 Espoo, Finland

**Keywords:** fused filament fabrication, cellulose, biopolymer, 3D printing, filament extrusion, tensile properties, fracture

## Abstract

Cellulose is an abundant and sustainable material that is receiving more and more attention in different industries. In the context of additive manufacturing, it would be even more valuable. However, there are some challenges to overcome in processing cellulose-based materials. Therefore, this study used a new thermoplastic cellulose-based granulate to show its potential in filament extrusion and the fused filament fabrication printing process. Furthermore, the mechanical properties were investigated. It was shown that filaments with a suitable and uniform diameter could be produced. A parameter study for printing revealed that adhesion of the material on the bed and between layers was an issue but could be overcome with a suitable set of parameters. Tensile bars with different orientations of 0°, +/−45°, and 90° were printed and compared with injection-molded samples. It could be shown that different mechanisms (single strand breakage, shear failure) caused fracture for different printing orientations. In comparison with injection-molding, the printed parts showed lower mechanical properties (moduli of 74–95%, a tensile strength of 47–69%, and an elongation at break of 29–60%), but an improvement could be seen compared with earlier reported direct granule printing. The study showed that FFF is a suitable process for the new cellulose-based material to fabricate samples with good mechanical properties.

## 1. Introduction

Additive manufacturing technologies open many possibilities for a resource-efficient and flexible production of individualized products, spare parts, or prototypes [1,2]. Especially thermoplastic extrusion technologies such as fused filament fabrication (FFF) or direct granule/pellet extrusion, have the advantage that they just need as much material as the part itself needs. It is unnecessary to manufacture a mold, and the need for post-processing is very low [3]. As these technologies are quite new and the requirements for 3D printing differ from the standard extrusion, new materials must be developed. A trend in additive manufacturing is that materials should not only have a good performance regarding printing and product properties but should also be environmentally friendly. The aim is to use biopolymers and biobased fillers to make the technology even more sustainable [4]. The most frequently used polymer is polylactic acid (PLA). It is based on natural resources such as corn, sugar cane, or potatoes [5]. PLA has low glass transition and melting temperatures and low shrinkage due to its amorphous structure. Therefore, it is easy to print via various affordable printers and open for mass consumption. However, PLA has drawbacks, such as low heat stability and high brittleness [6]. Composites and blends of PLA can overcome some of these disadvantages, but the low deformation temperature remains an issue [5,7]. Other possibilities for biopolymers in FFF are poly-(2-caprolactone) (PCL) or poly-(hydroxyalkanoates) (PHA) [6,8].

One biomaterial that is receiving more and more attention is cellulose. It is the most abundant polymer in nature and, therefore, a good choice as a renewable resource. In the past, the industry used cellulose as paper, foils, or coatings [9]. Previous processing methods were often based on cellulose solution in different solvents and afterward agglomeration/coagulation of the material into fibers or films. Prominent examples are fibers from regenerated cellulose such as lyocell or rayon [9,10,11]. In recent times there has been a growing demand for processing as melt. However, cellulose itself is not meltable because of high intermolecular forces based on hydrogen bonds leading to a theoretical melting point above its degradation temperature [9]. Some approaches used only cellulose fiber or cellulose nanomaterials in other matrix polymers, such as PLA [5,12,13] or PP [14]. Another study used the hemicellulose part of natural products for printing [15]. A method to use cellulose as a meltable thermoplastic material is the modification of cellulose to cellulose acetate, which is meltable. There are different possibilities to exchange OH-groups with ester groups such as acetate, propionate, or butyrate [9]. However, it is still quite complicated to process the material. The main reason is that there is only a small processing window between the softening and degradation temperature of cellulose acetate. Therefore, further modifications such as plasticizers are used to improve the flowability of the cellulose [16]. Some research has been done on all-cellulose composites, but still, the knowledge is limited [11,17].

When it comes to additive manufacturing, there are additional challenges. As the technology is a layer-by-layer process, the adhesion between the different layers is crucial for the mechanical performance of the part. For better welding, a higher temperature is favorable [18]. In the case of cellulose acetate, there is the beforementioned limitation by the degradation temperature. Further challenges are the adhesion on the printing platform, porosity in the part and warpage, as well as shrinkage [19]. In a previous study, different variations of cellulose-based thermoplastic materials were used as printing materials to evaluate processing and mechanical properties to identify suitable materials for the printing process. It was shown that cellulose esters with longer aliphatic carbon chains (≥8 C) could be used as plasticizers for cellulose acetate propionate (CAP) based compounds, and there is potential for improved material properties in printed materials [20]. A following study showed the recyclability of these materials up to seven processing cycles and improved performance in up to five times recycled materials [21]. In both studies, direct extrusion from granules was used. 

However, as FFF is widely incorporated in the 3D-printing community, both industrial and consumer, this study will focus on filament-based technology. The processing step of filament extrusion brings several challenges with it. The filaments need a suitable and uniform diameter for continuously feeding the filament during the printing process, providing a constant amount of material at the nozzle. Furthermore, mechanical strength during pulling and flexibility for winding are necessary. The filament should not have pores or a high ovality to ensure reproducible printing results. These aspects are rarely investigated in current literature [22]. There are a few biopolymer-based publications dealing in detail with the processing and characterization of filaments [23,24,25]. Focusing on cellulose, filament extrusion is reported for PLA-cellulose composites [13,26] or PP-cellulose-composites [12,14]. In contrast, filament extrusion and FFF printing of materials with a cellulose-based matrix have not been reported so far.

Therefore, this study investigates the processability of one selected cellulose-based material in filament extrusion and the following FFF printing process. Processing parameters are optimized in both steps to gain good-quality filaments and prints. Furthermore, the tensile properties of cellulose-based samples were investigated depending on printing orientation (0°, +/−45°, 90°) and compared with injection-molded samples.

## 2. Materials and Methods

### 2.1. Material

A cellulose-based granulate material was used. The material called 31NOVUMC16TC4V20-5 is a mixture of cellulose acetate propionate CELLIDOR CP300-13 (Albis Plastics GmbH, Hamburg, Germany) containing 13% commercial phthalate-free plasticizer, microcrystalline cellulose fibers VIVAPUR 105 (JRS Pharma GmbH, Weissenborn, Germany) and additives for better processability and fiber-polymer coupling. It contains 4% cellulose palmitate (VTT, Espoo, Finland) as an additional plasticizer and 1% epoxidized linseed oil Lankroflex™ (Valtris Specialty Chemicals, Independence, Ohio, USA) for improved fiber polymer coupling. Compared with other cellulose-based materials, it has a high cellulose-based carbon content of 50–60 wt%. VTT developed the material in the European research project “Novum project.” This particular material was chosen based on previous research on different cellulose materials [20]. Compounding for granules was made using a corotating twin-screw extruder (Berstorff ZE 25x33 D, Berstorff GmbH, Hannover, Germany). The extruder zone temperatures ranged from 80 °C to 205 °C, speed 100 rpm, and output of 3 kg/h.

### 2.2. Processing

**Figure 1:**
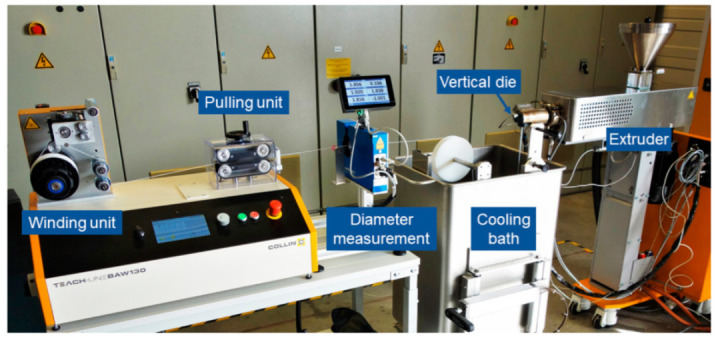
Filament extrusion setup (extrusion from right to left).

The granules were processed to filaments via an extrusion process. A single screw extruder Collin Teach-Line E20 (COLLIN Lab & Pilot Solutions, Meithenbeth, Germany) with a vertical die was used. The filament was cooled in a water bath and subsequently hauled off by a belt-pulling unit while a laser-based device measured the diameter. The complete setup is shown in Figure 1. A diameter of 1.75 mm was aimed. The screw speed was fixed at 45 rpm. The temperatures of the heating elements and the pulling speed were varied to achieve a round and uniform filament with a suitable diameter.

3D printing of the filament was done by FFF at a Raise3D Pro2 (Raise3D, Irvine, CA, USA) with a brass nozzle of 0.4 mm diameter. A BuildTak plate was used as a printing bed. The layer thickness was 0.15 mm, and the infill was 100% for all prints. Small geometries (50 mm × 10 mm × 2 mm) were used for parameter studies. Nozzle and bed temperature, printing speed, flow, and fan settings were adjusted. After the study was completed, the best settings were used to print 1A tensile bars (ISO 527) with line infill in different orientations: 0°, +/−45°, and 90° (at least six tensile bars each).

For later comparison, tensile bars were also produced by injection molding. An injection molding machine, Arburg 470 H1000-170 (Arburg, Loßburg, Germany), was used. Die and mold temperatures were 215 °C and 70 °C, respectively. The injection-molded parts were taken as a defect-free benchmark to evaluate the mechanical capability of the material and differences in printed pore-containing parts.

The material was dried at 80 °C in a vacuum oven overnight prior to all processing steps.

### 2.3. Characterization

Different characterization methods were used to determine the properties of the compound, the filaments, and the printed parts. 

At first, the processing window of the cellulose-based granulate was investigated. Differential scanning calorimetry (DSC) was used to observe the melting behavior. Two heating and one cooling cycle were carried out at a DSC 1 (Mettler Toledo, Columbus, OH, USA) with a heating/cooling rate of 10 °C/min in a temperature range of 25–250 °C. Two measurements were done. 

Furthermore, shear rheology was measured at an RDA3 of Rheometric Scientific (Piscataway, NJ, USA). Round specimens (25 mm diameter × 2 mm thickness) were prepared using a hot press. With a temperature ramp test, the region of good flowability of the material was determined. A frequency of 1 Hz, a strain of 10%, a heating rate of 4 °C/min, and a temperature range of 140–240 °C was used. Both DSC and rheology were done in an N_2_ atmosphere.

Furthermore, the water uptake of the material was investigated, measuring the weight of the granules. The material was dried at 80 °C in a vacuum oven overnight. Afterward, it was stored at lab conditions of 23 °C and 50% humidity. The weight was measured with a Standard Analytical Balance AG245 (Mettler Toledo, Columbus, OH, USA).

The tensile properties of the printed parts were determined according to ISO 527 using a universal testing machine, Zwick 1455 (ZwickRoell GmbH & Co. KG, Ulm, Germany). The test speed was 1 mm/min to determine Young’s modulus and 50 mm/min for subsequent measurement. The printed and injection-molded tensile bars were tested using at least six tensile bars for each set. All samples were dried at 80 °C overnight, except for one set of +/−45°-printed samples. Those were stored at lab conditions (23 °C, 50% humidity) and compared with dry +/−45°-printed samples to investigate the influence of water uptake on the mechanical properties.

Morphology of the filaments and printed parts was analyzed using a light microscope DM6000 (Leica Microsystems, Wetzlar, Germany). Samples were embedded in epoxy resin and then polished.

A Wide-Area 3D Measurement System Head VR-5200 (Keyence Corporation, Osaka, Japan) was used to investigate the fracture surface of the tested tensile bars.

## 3. Results and Discussion

### 3.1. Determination of the Processing Window

**Figure 2:**
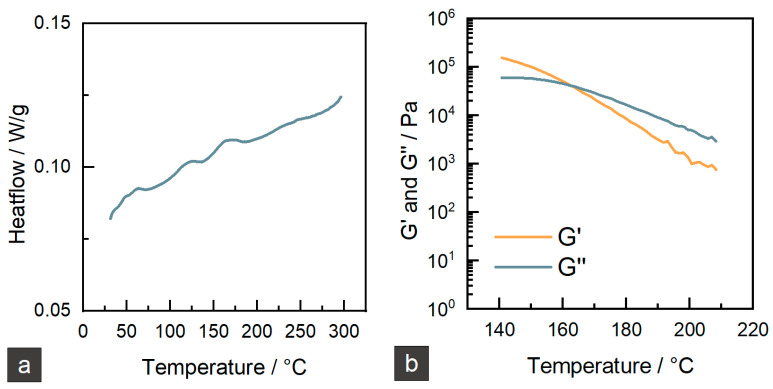
Thermal and rheological characterization of cellulose-based granules. (**a**) DSC, first heating curve, (**b**) shear rheological temperature ramp test.

To find a starting point for the processing temperatures of the new cellulose-based material, DSC should give the first hint on glass transition (*T_g_*) and melting points (*T_m_*). In Figure 2a, the result of the measurement can be seen. The curve shows three main steps at 57 °C, 116 °C, and 153 °C. As the material is a mixture of different components such as cellulose acetate propionate, cellulose microfibers, and plasticizers, these steps belong to their different *T_g_s*. A melting peak cannot be observed due to a non-regular substitution pattern of cellulose esters that prevents crystallization [9]. Further insight into the flow behavior of the cellulose material can be gathered with a rheological measurement. The results of a temperature ramp shear test are shown in Figure 2b. The curves are well defined until a temperature of 210 °C. Higher temperatures lead to very low viscosity and deviations from the measured values. There is a crossover point of G’ and G” at 163 °C. Usually, this point correlates with the melting point of a polymer. Even if the material has no real melting point, the crossover can be regarded as the start of flowability. Based on these investigations, 205–220 °C was taken as a start processing window for the subsequent extrusion trials. In this temperature range, good flowability is enabled while there is still some stability of the melt.

### 3.2. Filament Extrusion

Uniform diameter and homogeneity of the filament are two of the essential parameters in high-quality filament manufacturing. Differences in diameter will create an irregular flow rate that will affect the quality of FFF printed parts; likewise, lack of homogeneity on the filament due to unmolten particles of material or porosity will be detrimental when seeking high-quality FFF printing. The 3D printer used for this project employs 1.75 mm diameter filaments. As pretrials showed that too large diameters lead to a printing stop because of a blocked filament feeding, diameters slightly smaller than 1.75 mm were favored. Different processing parameters were tested, as shown in Table 1.

The first filament extrusion trial was promising as a consistent filament could be obtained with a diameter close to 1.75 mm. The mass temperature of the melt was 208 °C which was in the desired processing window. However, some irregularities could be seen through the filament with partially unmolten granules that created thicker diameter spots in the filament. The result of this first trial was a filament with a diameter of 1.85 ± 0.24 mm. Both the average diameter as well as the deviation extended the limit to be fed to the printer. To solve this problem, heating units were adjusted, and the temperature was increased to obtain a homogeneous, fully molten polymer. With the settings of filament 2, the mass temperature was raised to 221 °C. Higher temperatures could avoid the irregularities, but modifications had to be made to create a stable melt flow at the die. Furthermore, diameter deviations and particularly maximum diameters of more than 1.80 mm, should be avoided to ensure the filament will not become stuck in the Bowden tube while printing.

**Figure 3:**
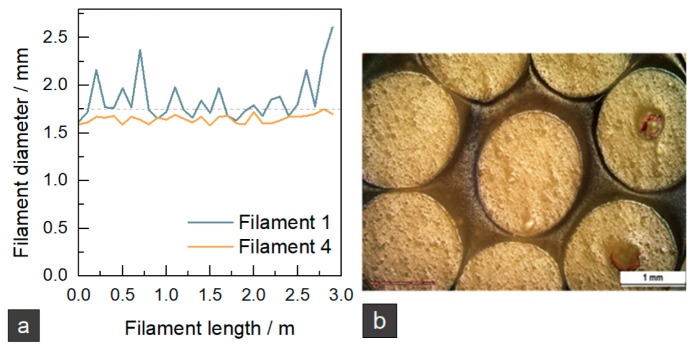
Results of filament extrusion. (**a**) Filament diameters of filament 1 and filament 4. The grey dotted line indicates the standard filament diameter of 1.75 mm. (**b**) Microscopic image of filament cross-section.

In two more steps, the final parameters of filament 4 were set as given in Table 1. The filament diameters of filament 1 and filament 4 are given in Figure 3a. It can be seen that the adjustment of processing parameters was successful so that a uniform filament with an average diameter of 1.65 ± 0.04 mm was produced. The shape and inner structure of the filament can be seen in Figure 3b. The filament has a smooth surface and shows little ovality. Small, randomly distributed pores can be detected in all filaments. In some spots, there are bigger pores in the middle of the filament. The rapid solidification of the filament could lead to shrinkage, but as the material is amorphous, the shrinkage should be low. Some red fiber-like spots can be detected, which could be related to the formation of more prominent pores. Currently, the reason is not fully clear and needs further investigation. 

It can be concluded that a filament with a suitable diameter and shape could be produced. It must be considered that the pores reduce the amount of feed material, which had to be compensated by suitable processing parameters in the printing process.

### 3.3. Fused Filament Fabrication

As the extrusion trials showed that a stable melt flow could be achieved with a die temperature of 210 °C, a standard PLA printing profile (see Table 2) was used as starting point for the printability study. The thermoplastic cellulose material showed good printability from the beginning with proper material deposition and high geometrical accuracy. However, parameters needed to be adjusted to tackle several challenges.

On the one hand, under-extrusion occurred between extruded paths. Gaps were present between adjacent extrusion layers. This phenomenon was due to too high a printing speed and insufficient flow rate (due to filament diameter being thinner than 1.75 mm and the porosity of the filament). The printing speed was reduced, and the flow rate was increased. Furthermore, adjustments regarding the temperature had to be made to achieve a lower viscosity and better melt flow. The nozzle temperature was increased gradually from 190 °C to 240 °C to improve the flowability of the material without degrading it. Above 240 °C, the material showed some signs of degradation such as color variations and the release of white color smoke during extrusion. Earlier work showed that critical temperatures regarding degradation occur in a range of 265–280 °C for different cellulose-based materials [20]. Therefore, it can be assumed that a short-term temperature influence of 240 °C will not damage the material.

**Figure 4:**
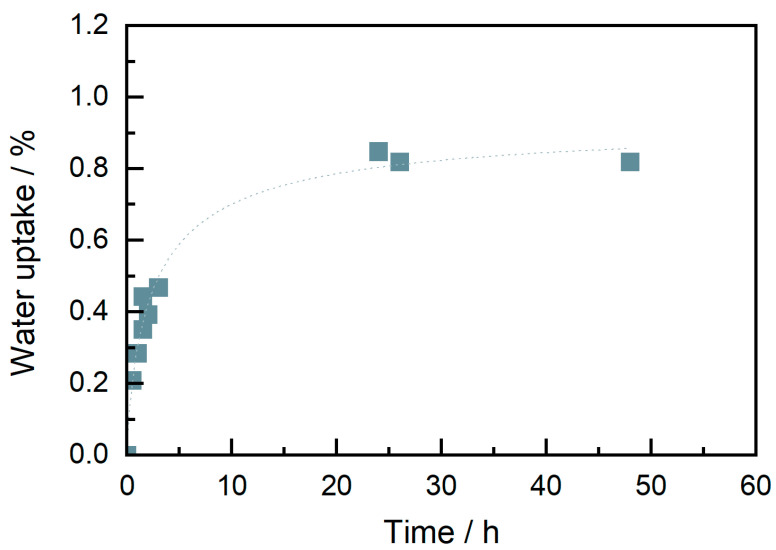
Water uptake of granulates at 23 °C, 50% humidity (dried before at 80 °C overnight).

**Figure 5:**
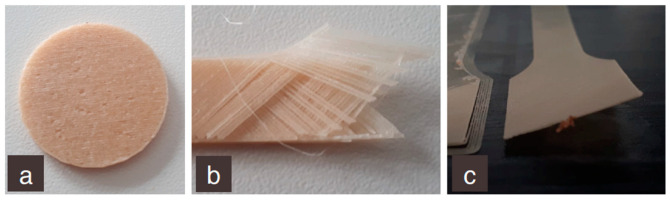
Printing challenges of cellulose-based filaments. (**a**) Porosity due to water uptake, (**b**) detachment of layers from each other, (**c**) detachment from printing bed.

On the other hand, the material showed a rapid water uptake, as shown in Figure 4. Even if the filament was dried before starting printing, after around four hours, water could be observed by bubble explosion sounds while the filament was being molten in the nozzle and by gaps in the extrusion paths (because of air bubbles) (Figure 5a). This problem was solved using a drying chamber with silica bags where the filament was stored during the print.

Another challenge was the low adhesion of adjacent layers in the z-direction (Figure 5b). Even if the test specimen showed good geometric accuracy and quality, the layers could easily be separated by little mechanical force. This problem was mainly due to the temperature of the nozzle. As said before, the polymer was printable and could flow at an initial nozzle temperature of 190 °C. However, this temperature was not high enough to fuse the layers effectively, and they could be separated easily. To improve the adhesion and the flowability, 240 °C was selected as the ideal nozzle temperature.

Related to the previous issue, material solidifying speed was another parameter that needed to be adjusted. During the first printing trials, the cooling fans were activated. This caused fast cooling and solidification of the printed layer, leading to bad welding to the next layer printed on top. To reduce the temperature difference and enable better diffusion and welding, the fans were deactivated during the printing process.

Adherence to the building plate also had to be improved as warping could be observed when printing large models due to the ineffective sticking of the part to the bed (Figure 5c). Initially, glue was used to improve adhesion. However, this solution was unproductive as the building plate needed to be cleaned with isopropanol and recalibrated after each printing. Furthermore, glue fragments generated an uneven surface underneath the first layer. However, the issue was solved by increasing the bed temperature from 60 °C to 90 °C and adding a brim.

Finally, the processing parameters in Table 2 were chosen as optimum parameters. 1A tensile bars were printed with infill directions of 0°, +/−45°, and 90° (with one wall layer).

In comparison with previous work, where direct granule printing and a nozzle with 0.8 mm were used, the FFF process with a nozzle size of 0.4 mm led to better geometrical accuracy. The single strands and layers were less visible than in a direct granule printing [20,21].

### 3.4. Mechanical Properties

**Figure 6:**
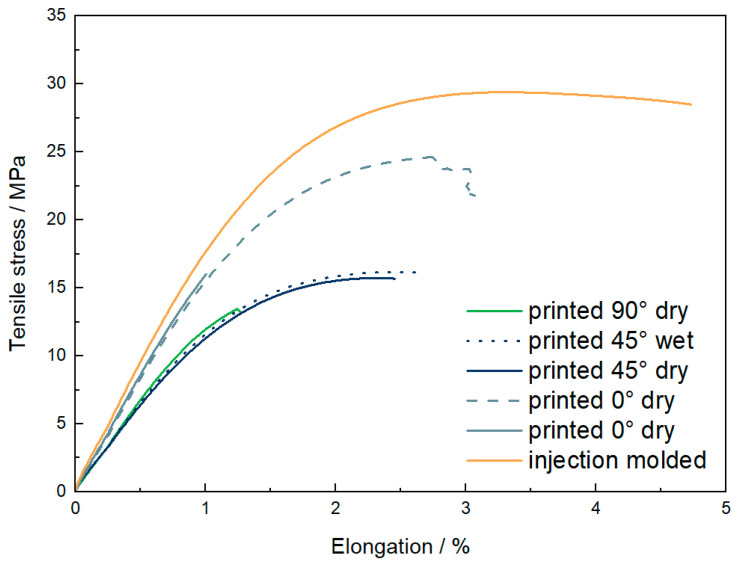
Stress-strain curves of tensile test of samples with different processing parameters and in different conditioning states (dry/wet).

The mechanical properties measured by tensile tests are given in Table 3. Additionally, one representative stress-strain curve for each set is shown in Figure 6 (two curves are shown for 0° orientation due to high deviations, which will be discussed later). Three aspects are discussed here: the influence of water, the influence of printing orientation, and the comparison of printed and injection-molded samples. 

At first, printed samples with a printing orientation of +/−45° were tested in a dried and conditioned (23 °C, 50% humidity) state. Young’s modulus, tensile strength, and elongation at break show no significant difference between dried and wet samples. Furthermore, the curves in Figure 6 also show the same shape. Therefore, it can be concluded that water uptake is a severe issue during processing but less critical for the tensile properties of the final part. Therefore, further discussions will be only based on dried tensile bars.

**Figure 7:**
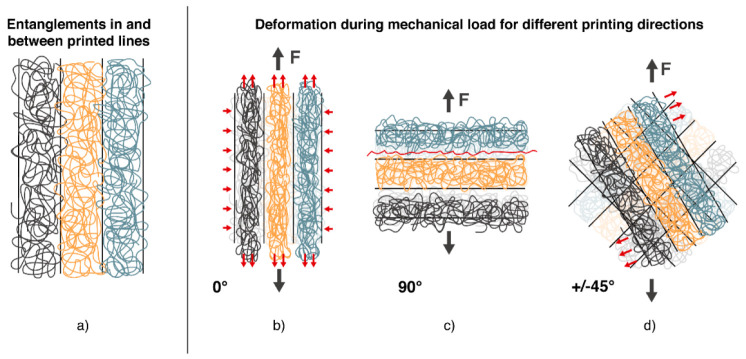
Schematic drawing of entanglements between printed strands/layers and influence on deformation under mechanical load of different infill printing orientations. (**a**) General build-up of layer structure, (**b**–**d**) Deformation of sample and single layers under tensile load depending on the printing orientation (0°, 90°, +/−45°).

The following comparison was made between different printing orientations. It can be seen that Young’s modulus of samples with a printing orientation of 0° is 1580 MPa and, therefore, around 25% higher than for samples with a printing direction of +/−45° (1240 MPa) and 90° (1250 MPa). As the lines are orientated in the loading direction for 0° samples, the load can be directly taken by the single strands without needing a load transfer from one strand to another. The moduli of samples printed in +/−45° and 90° directions are very close to each other, even if a higher modulus for the +/−45° direction would be expected due to the higher degree of orientation in the loading direction. Another effect seems to be dominating here, illustrated in Figure 7. As shown in 7a, the strands of one printed layer are aligned next to each other. Inside one strand, there are many entanglements of the polymer chains. In contrast, only a few entanglements are found between the strands. In 0°-direction (7b), the load is basically on the single strands, and transfer is not crucial. For 90° orientation (7c), it is clear that fusing areas with a low number of entanglements will be prone to deformation. Therefore, the resistance against deformation is lower, and Young’s modulus decreases. In the +/−45°-direction (7d), the adhesion between strands still is crucial. The single strands could be seen as fibers. When the load is applied, there is a demand to turn into the loading direction, as shown in Figure 7d. Therefore, Young’s modulus is based on the interaction between strands, leading to a similar modulus in 90°-direction.

**Figure 8:**
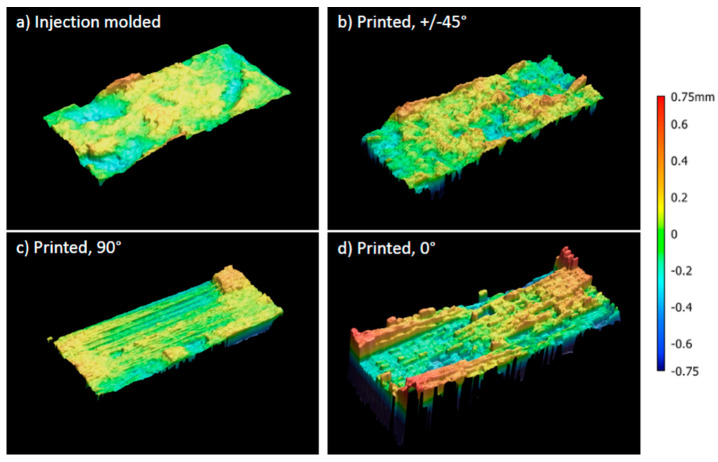
Fracture surfaces of tested tensile bars captured with optical analysis. (**a**) Injection molded, (**b**) printed with lines in +/−45°-orientation, (**c**) printed with lines in 90°-orientation, (**d**) printed with lines in 0°-orientation.

Looking at the tensile strength, the dominating aspects are defects leading to fracture. As printed samples always show porosity, these pores can be spots of stress concentrations where failure will start. The highest tensile strength is measured for 0°-orientation with 19.3 +/− 4.4 MPa. To explain the high standard deviation, it is worth looking at two different curves in Figure 6 and the fracture surface shown in Figure 8. All samples show a similar stress-strain curve in the linear-elastic region, but failure occurs at different maximum stresses between 15 and 25 MPa. The picture of the fracture surface reveals the single printed strands (Figure 8d). It can be concluded that sample failure is based on the sequence of single-strand breakage. In some cases, the printed strands fail simultaneously, and fracture occurs early. In other cases, a bridging effect leads to higher tensile strength and higher elongation at break. In contrast to the 0°-orientation, 90°-orientation shows the lowest tensile strength of 13.4 MPa and elongation at a break of 1.0%. On the fracture surface in Figure 8c, the strands can also be seen. Due to the 90°-orientation, there are bigger pores in the fracture plane, leading to higher stresses and earlier fracture. Bridging effects will not occur. With +/−45° orientation, tensile strength is 16.7 MPa and so in between the values of 0° and 90° infill direction. Elongation at break shows the highest value of 2.7%. A mix of different effects causes the failure here. Single strands are prone to turning, which causes shear stresses. As one strand is connected to many different strands, there can be some bridging effects accompanied by a single-strand deformation. The fracture surface is rough, as shown in Figure 8b, and the classical printing pattern can only be seen at the edges. This supposes a good adhesion and supports the theory that a mixed failure occurred.

Finally, the results of printed tensile bars were compared with those of injection-molded samples. The Young’s modulus of the injection-molded samples was 1670 MPa, which could almost be reached by the 0°-printed tensile bars. The modulus for the other printing directions was 25% lower. As explained before, the lower number of entanglements in printed samples is the reason for that. The differences between injection molding and printing become more pronounced for elongation at break and tensile strength. Here a decrease of up to 50% in tensile modulus and up to 70% in elongation at break is measured. This is mainly caused by the pores in printed samples, which lead to stress concentrations and finally to failure. The fracture surfaces show that injection-molded breakage correlates the most with the +/−45°-printed samples. In both cases, the interaction of polymer chains occurs all over the sample while it is restricted to the interaction between single strands in 0° or 90°-printed samples.

To evaluate the potential of FFF printing in comparison to the earlier used direct granule printing, Table 4 compares the tensile testing results of this study with those of the previous publication [21]. The differences in the values from injection molding can be explained by different testing speeds. Considering the relative change of Young’s modulus, tensile strength, and elongation at break, it can be seen that the decrease of properties for 3D-printed samples is remarkably lower with the FFF process. There are different reasons for that behavior. At first, the printing temperature in this study was higher, causing a higher diffusion and better adhesion between the layers. Furthermore, the layer structure of the samples in this study was finer, as the nozzle size was only 0.4 mm (compared with 0.8 mm for direct granule printing). Therefore pores in between the layers are smaller, causing lower stress concentrations. In summary, it can be concluded that not the technology itself is crucial for the mechanical properties, but different processing parameters. Therefore, there is also a good possibility for improvement of direct granule printing.

## 4. Conclusions

This study used a meltable cellulose-based material for filament extrusion and FFF printing for the first time. Processing, as well as the resulting mechanical properties of printed parts, were investigated.

Based on thermal and rheological measurements, a melt temperature of around 210 °C was proposed as a good processing temperature for the cellulose-based material. During the filament extrusion trials, slightly higher melt temperatures of 220 °C were favorable for a good flow and uniform filament extrusion. A filament with an average diameter of 1.65 mm, good roundness, and low diameter deviations was successfully produced. The filament could be printed with a FFF printer. Challenges such as water uptake, bad adhesion, and porosity were overcome with the following printing parameters: nozzle temperature of 240 °C, bed temperature of 90 °C, flow of 110%, deactivation of fan, and use of a dried filament. 

Investigating the tensile properties, the influence of water, printing orientation, and comparison of printed and injection molded samples were considered. It was shown that water uptake is only critical for processing but does not influence the tensile behavior of the samples. Comparing the different infill printing directions, it was shown that 0°-orientation leads to the loading of single printed strands with low interaction of those strands. This results in high values of strength and modulus but also in high deviations of values. In the 90° and +/−45° direction, the interaction and entanglement between strands and layers are more important. As the welding is not perfect, strength and stiffness are lower than for the 0° direction. Unlike injection-molded samples, all printed samples show lower modulus, strength, and elongation at break. The reason is that pores act as defects, and interactions/entanglements show weak spots between different layers. The study showed that the chosen processing route improved the mechanical properties of printed samples in comparison to the previously used direct granulate extrusion process.

As next step thermoplastic cellulose materials with improved strength properties should be evaluated regarding their printability and investigations of the quality of filaments will be done will be done. It will also be a future challenge to optimize the material and temperature management during processing to enable improved adhesion without damage to the material.

## Figures and Tables

**Table 1:** Processing parameters of the filament extrusion.

	Filament 1	Filament 2	Filament 3	Filament 4
Inlet/°C	30	30	30	30
Heating zone 1/°C	150	160	160	160
Heating zone 2/°C	220	225	225	225
Heating zone 3/°C	210	225	225	225
Heating zone 4/°C	210	220	220	220
Heating zone 5/°C	200	215	210	210
Heating zone 6 (die)/°C	200	210	205	210
Pulling speed/m/min	13.00	12.50	12.75	12.75

**Table 2:** FFF printing parameters. Left: initial printing parameters. Right: optimized printing parameters for cellulose-based filaments.

	Initial Parameters	Optimum Parameters
Nozzle temperature/°C	190	240
Building plate temperature/°C	60	90
Printing speed/mm/s	60	30
Flowrate/%	100	110
Layer height/mm	0.15	0.15
Cooling fans	Activated	Deactivated

**Table 3:** Results of tensile tests of printed and injection-molded samples.

	Printed 0° Dry	Printed ±45° Dry	Printed ±45° Wet	Printed 90° Dry	Injection-Molded
Young’s modulus/GPa	1.58 ± 0.06	1.24 ± 0.09	1.28 ± 0.06	1.25 ± 0.03	1.67 ± 0.10
Tensile strength/MPa	19.3 ± 4.4	16.7 ± 1.7	16.4 ± 0.8	13.4 ± 1.0	28.1 ± 1.6
Elongation at break/%	1.8 ± 1.1	2.7 ± 0.3	2.5 ± 0.6	1.3 ± 0.1	4.5 ± 1.2

**Table 4:** Comparison of the change of tensile properties from injection molding to 3D-printing between the FFF process (this study) and direct granule printing (previous study, [21]).

		Young’s Modulus	Tensile Strength	Elongation at Break
This study (FFF)	IM	1.67 GPa	28.1 MPa	4.5%
	3D	1.28 GPa	16.4 MPa	2.5%
	Change of properties	−23%	−42%	−45%
Previous study (granule extrusion) [21]	IM	1.84 GPa	25.5 MPa	9.5%
	3D	0.83 GPa	11.6 MPa	3.2%
	Change of properties	−55%	−55%	−66%

## Data Availability

Not applicable.
